# Cholera Outbreaks in Nigeria Are Associated with Multidrug Resistant Atypical El Tor and Non-O1/Non-O139 *Vibrio cholerae*

**DOI:** 10.1371/journal.pntd.0002049

**Published:** 2013-02-14

**Authors:** Michel A. Marin, Cristiane C. Thompson, Fernanda S. Freitas, Erica L. Fonseca, A. Oladipo Aboderin, Sambo B. Zailani, Naa Kwarley E. Quartey, Iruka N. Okeke, Ana Carolina P. Vicente

**Affiliations:** 1 Laboratory of Molecular Genetics of Microorganisms, Oswaldo Cruz Institute (IOC), Oswaldo Cruz Foundation (FIOCRUZ), Rio de Janeiro, Brazil; 2 Department of Medical Microbiology & Parasitology, College of Health Sciences, Obafemi Awolowo University, Ile-Ife, Nigeria; 3 Department of Medical Microbiology and Parasitology, University of Maiduguri, Maiduguri, Nigeria; 4 Department of Biology, Haverford College, Haverford, Pennsylvania, United States of America; University of California San Diego School of Medicine, United States of America

## Abstract

**Background:**

The current millennium has seen a steep rise in the number, size and case-fatalities of cholera outbreaks in many African countries. Over 40,000 cases of cholera were reported from Nigeria in 2010. Variants of *Vibrio cholerae* O1 El Tor biotype have emerged but very little is known about strains causing cholera outbreaks in West Africa, which is crucial for the implementation of interventions to control epidemic cholera.

**Methodology/Principal Findings:**

*V. cholerae* isolates from outbreaks of acute watery diarrhea in Nigeria from December, 2009 to October, 2010 were identified by standard culture methods. Fifteen O1 and five non-O1/non-O139 strains were analyzed; PCR and sequencing targeted regions associated with virulence, resistance and biotype were performed. We also studied genetic interrelatedness among the strains by multilocus sequence analysis and pulsed-field gel electrophoresis. The antibiotic susceptibility was tested by the disk diffusion method and E-test. We found that multidrug resistant atypical El Tor strains, with reduced susceptibility to ciprofloxacin and chloramphenicol, characterized by the presence of the SXT element, and *gyrA* ^Ser83Ile^/*parC* ^Ser85Leu^ alleles as well CTX phage and TCP cluster characterized by *rstR* ^ElTor^, *ctxB*-7 and *tcpA* ^CIRS^ alleles, respectively, were largely responsible for cholera outbreaks in 2009 and 2010. We also identified and characterized a *V. cholerae* non-O1/non-O139 lineage from cholera-like diarrhea cases in Nigeria.

**Conclusions/Significance:**

The recent Nigeria outbreaks have been determined by multidrug resistant atypical El Tor and non-O1/non-O139 *V. cholerae* strains, and it seems that the typical El Tor, from the beginning of seventh cholera pandemic, is no longer epidemic/endemic in this country. This scenario is similar to the East Africa, Asia and Caribbean countries. The detection of a highly virulent, antimicrobial resistant lineage in Nigeria is worrisome and points to a need for vaccine-based control of the disease. This study has also revealed the putative importance of non-O1/non-O139 *V. cholerae* in diarrheal disease in Nigeria.

## Author Summary

Cholera is acute watery diarrhoea, severely dehydrating, caused by *Vibrio cholerae*, a bacterium ubiquitous in aquatic environments. Cholera is a global threat, particularly, in areas where sanitary conditions, such as drinking water and sewage, are not available. Seven cholera pandemics, all originating in Asia, occurred. The ongoing pandemic, the 7th, has been caused by *V. cholerae* El Tor biotype. Recently, El Tor has undergone genetic changes and the strains being referred to as “atypical” El Tor are rapidly replacing the original El Tor in many areas. The atypical El Tor is characterized by multi-antibiotic resistance and changes in the major virulence determinants. Cholera caused by atypical strains may be more clinically severe. In Africa, cholera outbreaks are occurring with increasing frequency and severity, as demonstrated by the recent major outbreaks in Nigeria, Angola, Mozambique and Zimbabwe. Here, we performed a comprehensive characterization of *V. cholerae* isolated from different recent outbreaks in Nigeria. Our results show that cholera outbreaks in Nigeria are driven by atypical El Tor strains, as worldwide.

## Introduction

*Vibrio cholerae* is a human pathogen that causes cholera, a severe acute watery diarrhea. There are more than 200 *V. cholerae* serogroups, however only O1 and O139 are responsible for most epidemics and pandemics of cholera. Serogroup O139 is restricted to some parts of Asia but serogroup O1, classified as El Tor and classical biotypes, was found worldwide [1], [2]. The classical biotype was responsible for severe clinical manifestation but the conventionally less virulent El Tor is better adapted to the environment [3].

The major virulence factors are the cholera toxin (CT) encoded by the *ctxAB* operon on the lysogenic bacteriophage CTXΦ [4], and the toxin-coregulated pilus (TCP) encoded by the VPI-I island, an essential factor for the colonization and also receptor for the CTXΦ [5]. Classical and El Tor biotypes can be distinguished by several genetic markers, such as *rstR*, *tcpA*, *ctxB* genes, the Vibrio seventh pandemic island-I (VSP-I) and VSP-II, and by the number of genes in the repeat toxin region (RTX) [2], [6].

Recently, *V. cholerae* strains have been identified showing markers of both classical and El Tor biotypes. These strains were assigned as hybrid biotype, altered or El Tor variants presenting a typical El Tor phenotype but with non-El Tor *ctxB* allele [2]. In contrast to *V. cholerae* from the beginning of 7^th^ cholera pandemic, these atypical biotype strains are frequently multidrug resistant. Changes in the antibiotic resistance profiles of *V. cholerae* are associated with mutations in housekeeping genes as well as acquisition of Integrative Conjugative Elements (ICEs) and other mobile elements [7], [8].

Africa is endemic for cholera and frequently affected by outbreaks and epidemics, but there are few molecular epidemiology studies characterizing the determinants of these episodes. Nigeria is in one of the three major current cholera foci in the world [9]. In 2009, outbreaks began in Nigeria and other countries at the Lake Chad basin [8] with the first reports coming from Maiduguri, a city in the far north-east of the country. Subsequently, outbreaks were reported from distant locales in Northern and Western Nigeria, and in 2010 a severe outbreak, which started in the Northern Nigeria spreading through the country, was projected as the worst outbreak in Nigeria since 1991. This outbreak was marked with highest case-fatality [10], what could be in part due to changes in *V. cholerae* infectivity even though the organism remains largely unknown. It can be hypothesized that an index strain has been disseminated cross-country by human travel.

Here, we performed a comprehensive characterization of representative *V. cholerae* strains from sequential outbreaks in Nigeria (Maiduguri/2009, Bauchi/2010 and Ile Ife/2010) by means of biotyping, multilocus sequence analysis (MLSA), pulsed-field gel electrophoresis (PFGE), as well as detection and sequencing of virulence related genes and genetic determinants of antimicrobial resistance. Our results show that recent cholera outbreaks in Nigeria are driven by atypical El Tor strains and we reported the presence of a non-O1/non-O139 lineage. The atypical El Tor strains showed one unique combination of virulence factor alleles and antimicrobial resistance to sulphonamides, trimethoprim/sulfamethoxazole, streptomycin, nalidixic acid and reduced susceptibility to ciprofloxacin and chloramphenicol.

## Materials and Methods

### Bacterial strains

Twenty *V. cholerae* strains (15 O1 and five non-O1/non-O139 strains) isolated from outbreaks in Nigeria and reference strains were analyzed (Table 1). We included in our analysis three isolates that originated from Nigeria in the beginning of the seventh pandemic (1970's), 16 isolates from three recent cholera outbreaks and one strain isolated from a water sample. *V. cholerae* isolates were identified and confirmed by standard culture methods at isolation during the epidemics and again prior to molecular characterization, and their serogroups and biotypes were identified by biochemical and molecular methods [11].

**Table 1:** Genetic and phenotypic characteristics of *V. cholerae* from Nigeria.

Strain	Serogroup (biotype)	Place of isolation	Year of isolation	Virulence related genes and biotypes					Related to antibiotic resistance						
				VSP-II		*ctxB* *	*tcpA*	*rstR*	IntI1	IntI2	SXT	Quinolone resistance		Antibiotic sensitivity	
				VC0511	VC0513							*gyrA*	*parC*	MIC^CIP^ (µg/ml)	MIC^CM^ (µg/ml)
Reference¥															
N16961	O1 (El Tor)	Bangladesh	1975	+	+	3	ET	ET	-	-	−	Ser83	Ser85	ND	ND
CIRS101	O1 (El Tor)	Dhaka, Bangladesh	2002	−	+	1	ET^CIRS^	ET	-	-	+	Ser83Ile	Ser85	ND	ND
MO10	O139	Madras, India	1992	−	+	3	ET	ET	-	-	+	Ser83	Ser85	ND	ND
O395	O1 (Classical)	India	1965	−	−	1	CL	CL	-	-	−	Ser83	Ser85	ND	ND
RC27	O1 (Classical)	Indonesia	1991	−	−	1	CL	CL	-	-	−	Ser83	Ser85	ND	ND
Nigeria strains															
VC79	O1	Nigeria	1971	+	+	-	-	ET	-	-	−	Ser83	Ser85	0.008	ND
VC111	O1	Nigeria	1972	+	+	3	ET	ET	-	-	−	Ser83	Ser85	0.008	ND
VC869	O1	Nigeria	1971	+	+	3	ET	ET	-	-	−	Ser83	Ser85	0.008	ND
VC832	O1	Maiduguri	2009	−	+	7	ET^CIRS^	ET	-	-	+	Ser83Ile	Ser85Leu	0.380	8.0
VC841	O1	Maiduguri	2009	−	+	7	ET^CIRS^	ET	-	-	+	Ser83Ile	Ser85Leu	0.250	8.0
VC833	O1	Ile Ife	2010	−	+	7	ET^CIRS^	ET	-	-	+	Ser83Ile	Ser85Leu	0.250	6.0
VC834	O1	Ile Ife	2010	−	+	7	ET^CIRS^	ET	-	-	+	Ser83Ile	Ser85Leu	0.250	6.0
VC835	O1	Ile Ife	2010	−	+	7	ET^CIRS^	ET	-	-	+	Ser83Ile	Ser85Leu	0.380	6.0
VC991	O1	Ile Ife	2010	−	+	7	ET^CIRS^	ET	-	-	+	Ser83Ile	Ser85Leu	0.380	6.0
VC997	O1	Ile Ife	2010	−	+	7	ET^CIRS^	ET	-	-	+	Ser83Ile	Ser85Leu	0.380	6.0
VC1001	O1	Ile Ife	2010	−	+	7	ET^CIRS^	ET	-	-	+	Ser83Ile	Ser85Leu	0.380	6.0
VC1007	O1	Ile Ife	2010	−	+	7	ET^CIRS^	ET	-	-	+	Ser83Ile	Ser85Leu	0.380	8.0
VC836	O1	Bauchi	2010	−	+	7	ET^CIRS^	ET	-	-	+	Ser83Ile	Ser85Leu	0.380	8.0
VC996	Non-O1	Bauchi	2010	−	−	-	-	-	-	-	−	Ser83	Ser85	0.023	0.5
VC998	Non-O1	Bauchi	2010	−	−	-	-	-	-	-	−	Ser83	Ser85	0.008	0.5
VC999	O1	Bauchi	2010	−	+	7	ET^CIRS^	ET	-	-	+	Ser83Ile	Ser85Leu	0.380	6.0
VC1004	O1	Bauchi	2010	−	+	7	ET^CIRS^	ET	-	-	+	Ser83Ile	Ser85Leu	0.500	6.0
VC1006	Non-O1	Bauchi	2010	−	−	-	-	-	-	-	−	Ser83	Ser85	0.012	0.5
VC1009	Non-O1	Bauchi	2010	−	−	-	-	-	-	-	−	Ser83	Ser85	0.008	0.5
VC1005§	Non-O1	Ile Ife	2010	−	−	-	-	-	-	-	−	Ser83Ile	Ser85Leu	0.250	ND

CL, classical; ET, El Tor; CIP, ciprofloxacin; CM, Chloramphenicol; ND, not determined;

* , *ctxB* genotype [16];

¥ , The genetic characteristics were determined by *in silico* analysis;

§ , VC1005 is an environmental isolate.

### PCR amplification and sequencing

PCR were performed targeting the genetic elements and regions associated with *V. cholerae* virulence, resistance and biotype. Virulence: VSP-II (VC0511, VC0513), VPI-I (*tcpA*), CTXΦ (*ctxB*), heat stable enterotoxin (NAG-ST), type three secretion system (T3SS), type VI secretion system (T6SS), enterotoxigenic hemolysin (*hlyA*) and RTX toxin (*rtxA*). Resistance: *IntI*1, *IntI*2 integrases and variable regions from class 1 and 2 integrons and the 3′ conserved sequence from class 1 integron, SXT element-integrase gene and associated SXT resistance genes (*floR*, *sul2*, *dfrA1* and *strAB*) and genes related to quinolone resistance (*gyrA*, *gyrB*, *parC* and *parE*). Biotype-specific repeat sequence transcriptional regulator (*rstR*), *ctxB* alleles and *rfb* gene specific for O1 serogroup 1 were also amplified. The nucleotide sequences of primers employed are listed in Table 2.

**Table 2:** PCR primers used in this study.

Primer	Sequence (5′-3′)	Reference
Housekeeping gene		
recA-01-F	TGARAARCARTTYGGTAAAGG	[43]
recA-02-R	TCRCCNTTRTAGCTRTACC	
pyrH-04-F	ATGASNACBAAYCCWAAACC	[43]
pyrH-02-R	GTRAABGCNGMYARRTCCA	
mdh-1	ATGAAAGTCGCTGTTATT	[44]
mdh-2	GTATCTAACATGCCATCC	
Biotype related genes		
Serogroup O1		
O1-F	GTTTCACTGAACAGATGGG	
O1-R	GGTCATCTGTAAGTACAAC	[11]
rstR		
rstRclaF	TTTGCTACTTCTTCTTGGTT	
rstRETF	TGAGCATAAGCTCTTGATTT	
rstAR	CCGTGAAAGTCATCAACG	[45]
Virulence gene primers		
VSP-II		
VC0511F1	CTTGCTGCGTACTTAGCA	
VC0511R1	AGTAGCATCGCTCTCGTA	[14]
VC0513F1	CTGAGGTGTTATATGTTTCG	
VC0513R1	TCAAATTTCCTGACAGTTCC	[14]
VPI-I		
TCPA1	CACGATAAGAAAACCGGTCAAGAG	
TCPA2	ACCAAATGCAACGCCGAATGGAGC	[46]
CTX Φ		
CT8	GCAGTCAGGTGGTCTTATTGC	
CT10	TCCAGATATGCAATCCTCAG	[35]
NAG-ST		
ST-1	GAGAAACCTATTCATTGCA	
ST-2	GCAAGCTGGATTGCAAC	[47]
T3SS		
vcsV2-F	GGCTCACCAGCTGTTATGGT	
vcsV2-R	CGTATTGCACAAGTAGCCGC	This study
T6SS		
vasH-F	TGTTGATGGGCGAGAGTCAC	
vasH-R	ACGTGTGTGGCAGATACCAG	This study
HlyA		
VC0489F	AGATCAACTACGATCAAGCC	
VC0489R	AGAGGTTGCTATGCTTTCTAC	[44]
RTX		
rtxA1	GCGATTCTCAAAGAGATGC	
rtxA2	CACTCATTCCGATAACCAC	[44]
Resistance gene primers		
Integron related primers		
INT1 F	AAAACCGCCACTGCGCCGTTA	
INT1 R	GAAGACGGCTGCACTGAACG	[13]
INT2 F	GCGTTTTATGTCTAACAGTCC	
INT2 R	AAGTAGCATCAGTCCATCC	[24]
SXT integrase		
SXTF	TCGGGTATCGCCCAAGGGCA	
SXTR	GCGAAGATCATGCATAGACC	[48]
ICE's related genes		
dfrA1 F	CGTGACAGGTTTGCGAATC	
dfrA1 R	ATGGAGTGCCAAAGGTGAAC	This study
strB F	CGTTGCTCCTCTTCTCCATC	
sul2 R	CGTCAACATAACCTCGGACA	This study
FloR F	GTGATTTTTGGTCCGCTCTC	
FloR R	TCGGTAGGATGAAGGTGAGG	This study
Quinolone resistance		
gyrA_vF	AATGTGCTGGGCAACGACTGG	
gyrA_vR	GTGCGCGATTTTCGACATACG	[49]
gyrB_vF	GGAAATGACTCGCCGTAAAGG	
gyrB_vR	GTTGTGATAACGCAGTTTATCTGGG	[49]
parC_vF	GTCTGAGTTGGGTCTCTCGGC	
parC_vR	AGAATCTCGGCAAACTTTGACAG	[49]
parE_vF	ATGCGTGCCAGCAAGAAAGTG	
parE_vR	TTATCGCTGTCAGGGTCAATCC	[49]
oqxA_F	CTCGGCGCGATGATGCT	
oqxA_R	CCACTCTTCACGGGAGACGA	[50]
qepA_F	GCAGGTCCAGCAGCGGGTAG	
qepA_R	CTTCCTGCCCGAGTATCGTG	[51]
qnrA_F	TTCAGCAAGAGGATTTCTCA	
qnrA_R	GGCAGCACTATTACTCCCAA	[52]
qnrB_F	CCTGAGCGGCACTGAATTTAT	
qnrB_R	GTTTGCTGCTCGCCAGTCGA	[52]
qnrS_F	CAATCATACATATCGGCACC	
qnrS_R	TCAGGATAAACAACAATACCC	[52]
qnrVC_F	AATTTTAAGCGCTCAAACCTCCG	
qnrVC_R	TCCTGTTGCCACGAGCATATTTT	[53]
aac(6′)-lb-cr _F	TTGCGATGCTCTATGAGTGGCTA	
aac(6′)-lb-cr _R	CTCGAATGCCTGGCGTGTTT	[54]

### MLSA

Amplification and sequencing of the *pyrH*, *recA* and *mdh* housekeeping genes were performed as described previously (Table 2). Sequence alignments and phylogenetic analyses were conducted using MEGA5 [12], based on the minimum evolution method using concatenated sequences. Distance estimations were obtained by Kimura two-parameter model. Two thousand bootstrap replicates were performed.

### Pulsed-Field Gel Electrophoresis

DNA macrorestriction profile was obtained using *NotI* enzyme according to procedures described elsewhere [13]. The PFGE dendrogram was constructed using BioNumerics software (Applied Maths, Belgium). The similarity was determined by Dice coefficient and cluster analysis was carried out with the unweighted-pair group method using average linkages (UPGMA).

### Disk diffusion susceptibility testing and Etest

Antibiotic susceptibility testing was performed by the disk diffusion method, with Muller-Hinton Agar (Oxoid, Basingstoke, UK), according to Clinical and Laboratory Standards Institute (CLSI, 2010) standards. Strains were tested for resistance to: Nalidixic acid, Ampicillin, Cephalothin, Cefpirom, Cefoxitin, Cefuroxime sodium, Ciprofloxacin, Chloramphenicol, Erythromycin, Spectinomycin, Compound sulphonamides, Streptomycin, Sulphamethoxazole, Sulphamethoxazole-Trimethopim, Trimethopim and Tetracycline. Minimum Inhibitory Concentrations (MICs) for Ciprofloxacin and Chloramphenicol were determined with the E-test (bioMerieux, Marcy l'Etoile, France).

## Results

### *V. cholerae* genotyping

We determined the genetic relationships among *V. cholerae* strains consisting of Nigeria strains from 2009/2010 and 1971/1972, one environmental isolate (2010) and well-characterized reference strains. A MLSA tree based on concatenated sequence of *recA* (666 bp), *pyrH* (456 bp) and *mdh* (591 bp) gene fragments were constructed.

**Figure 1:**
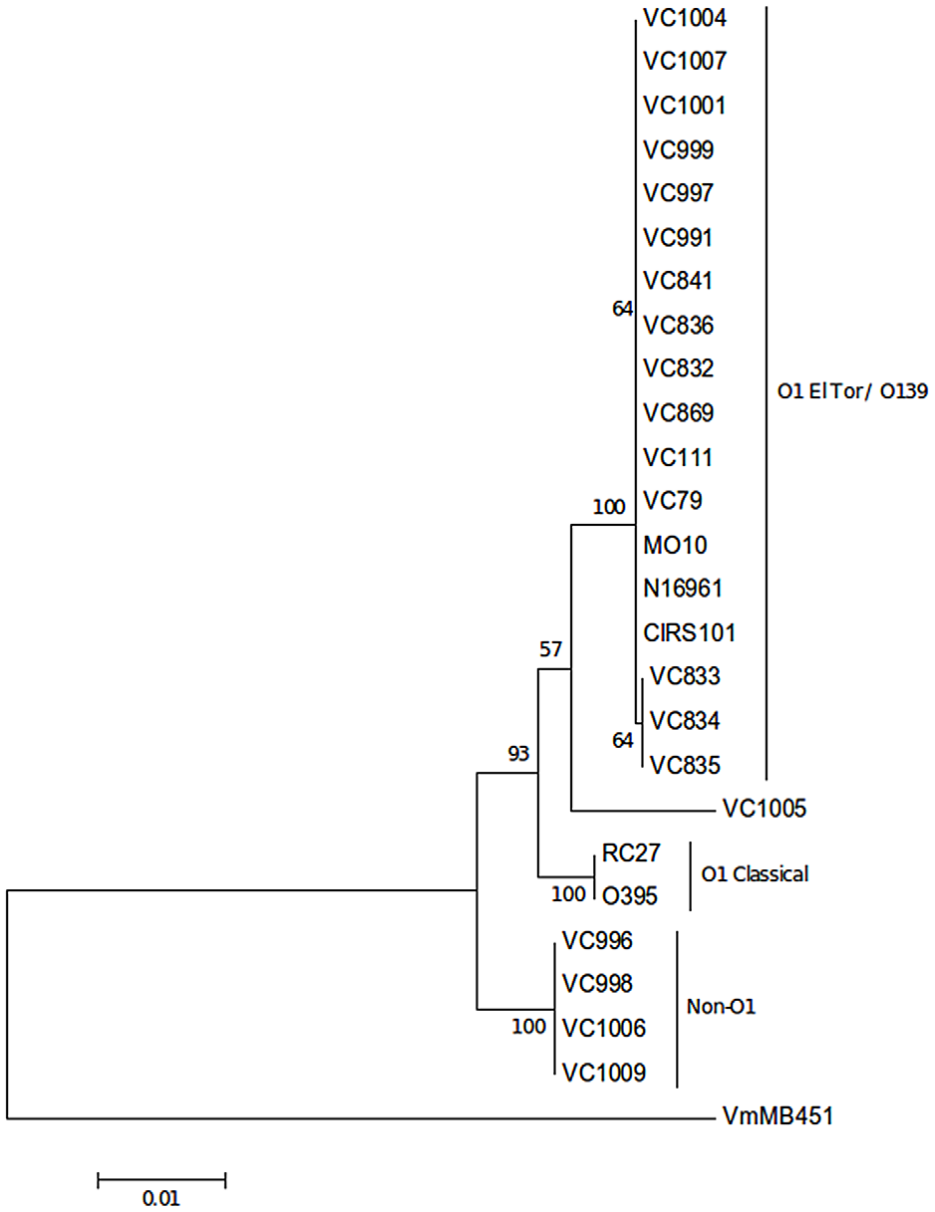
Genetic relatedness of Nigeria *V. cholerae* strains. The epidemic O1 Nigeria strains are within the El Tor/139 cluster and the Non-O1/Non-O139 strains characterized a distinct cluster. This Minimum Evolution tree was constructed based on Kimura-two-parameter method using the concatenated nucleotide sequences of *rec*A, *pyr*H and *mdh* gene fragments. Two thousand bootstrap replicates were performed; bootstrap values are given at the nodes. *Vibrio mimicus* MB451strain was used as outgroup.

**Figure 2:**
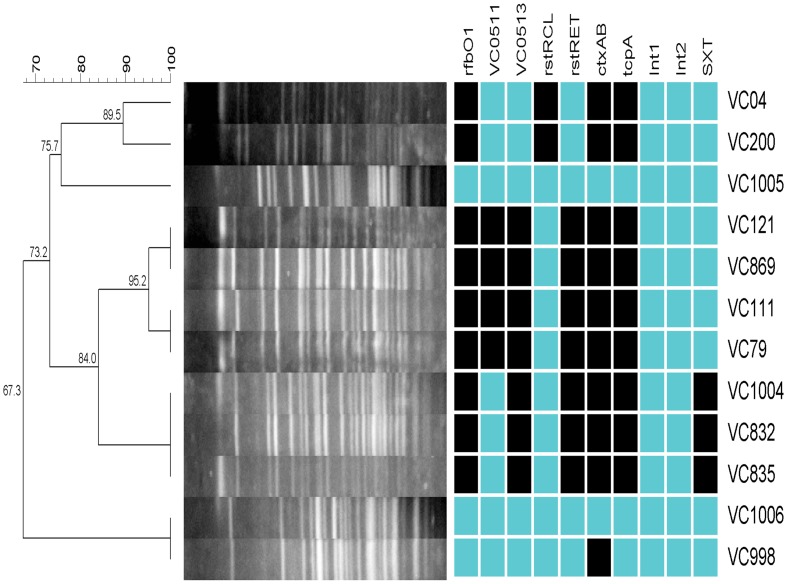
Dendrogram of PFGE patterns of representative *V. cholerae* O1 and Non-O1/Non-O139 strains. The similarity was determined using the Dice coefficient, and cluster analysis was performed using the UPGMA. The right panel shows the presence (dark box) and absence (light box) of genomic traits. VC04 and VC200 belong to the classical lineage; VC121 is an India strain from the beginning of the 7^th^ cholera pandemic.

The MLSA tree showed three major clusters: (i) O1 El Tor, O139 (MO10 strain) and the O1 clinical Nigeria strains, including the ones from the 1970s (Figure 1), (ii) O1 classical (O395 and RC27) and (iii) non-O1/non-O139 Nigeria strains. The average pairwise difference for the *V. cholerae* concatenated sequences was 1.5%. The divergence between the El Tor/O139 cluster and the classical cluster was 1.2% while the El Tor/O139 cluster and non-O1 cluster was 1.9%. This grouping was also revealed by macrorestriction analysis using representative strains of the MLSA analysis (Figure 2. VC04 and VC200 strains were used as representative classical strains of the 6^th^ pandemic and El Tor VC121 of the beginning of the 7^th^ pandemic from India. The PFGE profiles showed that the Nigeria strains from the beginning of the 7^th^ pandemic (VC869, VC111, VC79) and the recent strains (VC1004, VC832 and VC835) are genetically related and shared a profile with El Tor VC121. The non-O1 strains (VC1006 and VC998) shared a unique profile distinct from the O1 strains.

### Biotyping and virulence gene typing

PCR-verified serogroup analysis demonstrated that 15 of the 20 strains studied, belonged to serogroup O1 and five were non-O1 isolates (Table 1). *V. cholerae* O1 strains are classified into El Tor, classical and atypical El Tor [2] based on *tcpA* (toxin co-regulated pilin A), *ctxB* (cholera toxin B), *rstR* (repeat sequence transcriptional regulator), as well as variations in VSP-II (Vibrio seventh pandemic island II). The VSP-II gene cluster characterizes the El Tor strains, and comprises a region of ∼24 ORFs. In this study, *V. cholerae* O1 strains were genotyped based on sequence information of *rstR*, *ctxB*, *tcpA*, VSP-II regions (locus VC0511 and VC0513).

We screened for the presence of two loci of the VSP-II, VC0511 and VC0513, which are considered the most variable loci in the VSP-II island [14], [15]. The Nigeria strains from 1970's and the prototypical N16961, were positive for VC0511 and VC0513 loci, while the more recently recovered 2009/10 O1 El Tor Nigeria isolates harbor only VC0513 (Table 1), indicating that these lineages carry a different version of VSP-II than earlier isolates. The current non-O1 Nigeria strains were negative for both, VC0511 and VC0513 loci.

The *ctxB* present in all Nigeria current O1 El Tor strains belong to the atypical genotype 7, characterized by the non conservative change His20Asn [16]. El Tor strains from the beginning of the 7^th^ pandemic in Nigeria, as worldwide, were characterized by the canonical El Tor *ctxB* genotype 3 and keep their own repressor *rstR* ^ElTor^ gene of the CTX prophage.

Concerning the *tcpA* alleles, there are *tcpA* ^ElTor^, *tcpA* ^classical^ and *tcpA* ^NonO1^ disseminated among *V. cholerae* strains. All current O1 Nigeria strains harbor *tcpA* ^CIRS^ allele (Table 1), recently described in the CIRS101 strain, a *V. cholerae* O1 El Tor from Bangladesh, 2002 [17]. Based on these genotypes, the current epidemic *V. cholerae* O1 strains from Nigeria are classified as atypical El Tor biotype.

Although, the current non-O1/non-O139 Nigeria strains were negative for *rstR*, *ctxB*, *tcpA*, VSP-II regions (locus VC0511 and VC0513) as well NAG-ST and T3SS (*vcsV2* gene), they are positive for T6SS (*vasH* gene), *hlyA* and *rtxA* genes, all virulence associated factors involved in diarrheiogenic property [18]–[21]. Therefore, a non-O1/non-O139 lineage harboring a set of virulence determinants could be associated with a cholera-like diarrhea [22].

### Characterization of elements associated with antibiotic resistance

Antibiotic multidrug resistance is becoming increasingly common among the atypical *V. cholerae* strains, mostly associated with acquisition of genes and/or modification in the antibiotic target genes [23]. According to our results, the current O1 Nigeria strains were resistant to streptomycin, trimethoprim and sulfonamides. In *V. cholerae*, these resistances, are frequently associated with class 1 and 2 integrons [24] and SXT element, which is a *V. cholerae*-derived ICE (integrating and conjugative element) [25]. Thus, we investigated the presence of these elements in the Nigeria strains. All the current O1 strains harbor an ICE element, determined by the presence of the SXT integrase gene, contrasting with non-O1 and the O1 from the 1970's (Table 1). No evidence was found for the presence of class 1 and 2 integrons (which we sought by PCR for *intI*1 and *intI*2, respectively. Table 1). We performed PCR targeting ICE associated genes *floR*, *sul2*, *dfrA1*, and *strAB*, associated with chloramphenicol, sulfamethozaxole, trimethoprim and streptomycin resistance, respectively [7], [26]. All these genes were identified, explaining the resistance profile, including reduced susceptibility to chloramphenicol, of the current O1 strains.

Majority of the 2009/10 Nigeria O1 strains showed reduced susceptibility to ciprofloxacin (Table 1) as well as resistance to nalidixic acid. Quinolone resistance has been attributed to mutations in *gyrA* (Ser83Ile) and in *parC* (Ser85Leu) [26], [27] and these alleles were found in these strains. We additionally screened the 2009/10 isolates for horizontally transmitted quinolone-resistance genes *qnrA*, *qnrB*, *qnrS*, *qnrVC*, *qepA*, *oqxAB* and *aac(6′)-Ib-cr*. The isolates were all negative for all of these targets. None of these resistance phenotypes or genotypes detected in the 2009/10 isolates were found in strains isolated in Nigeria during the1970's. Additionally, resistance phenotype and genes were not seen in the 2009/10 non-O1/non-O139 isolates, with the exception of the environmental non-O1/non-O139 VC1005 strain, which showed resistance to nalidixic acid attributable to mutations in *gyrA* and *parC* (Table 1).

## Discussion

Since 1995 and over a period of more than a decade the canonical El Tor has been replaced in Kolkata, India by atypical El Tor. They were originally described in South Asia [2], [28], [29] but recent reports have shown their spread to all continents, reaching even Mexico [30] and Haiti [26]. Based only on *ctxB* genotyping, atypical El Tor have already been reported in some African countries, where cholera has become a serious public health threat in recent years [7], [8], [31]–[35].

Despite the great impact from cholera, few investigators focused on the molecular epidemiology of *V. cholerae* in West Africa. A recent genomic survey of 154 isolates worldwide did not include any isolates from West Africa [36]. Due to the plasticity of *V. cholerae* resulting in the constant emergence of variants, surveillance and characterization of outbreak strains, and their antibiotic resistance determinants, is essential to defining the complex scenario of cholera in this continent as well as worldwide. Recently, it was shown that different *V. cholerae* O1 lineages were responsible for cholera outbreaks in Ghana [35]. Here, we provide evidence that different *V. cholerae* lineages are driving cholera outbreaks in Nigeria.

Molecular typing of 20 *V. cholerae* strains from Nigeria, allowed us to identify atypical O1 strains as well as a non-O1/non-O139 lineage. To date, there are only two studies performing molecular characterization of *V. cholerae* strains causing cholera in Nigeria and very few microbiological outbreak investigations [8], [17]. Both molecular studies focused on the allelic characterization of the two major virulence genes and one of them addressed also the quinolone resistance determinants. Talkington *et al*, who analyzed two 2008 isolates from Nigeria, reported two profiles: *ctxB*-1/*tcpA* ^ElTor^/*rstR* ^ElTor^ and *ctxB*-1/*tcpA* ^CIRS^/*rstR* ^ElTor^ [17]. Quilici and collaborators analyzing ten strains from 2009 showed a unique allelic profile: *ctxB*-7/*tcpA* ^ElTor^/*rstR* ^ElTor^ [8]. They reported that these isolates were identical to nine isolates from neighboring Cameroon, which also borders the Lake Chad basin. These isolates are temporally and geographically proximal to the 2009 Maiduguri outbreak from which we obtained isolates VC832 and VC841 for our study. In our study, considering these set of genes, the 12 *V. cholerae* O1 strains, from 2009/2010 cholera outbreaks including the 2009 Maiduguri outbreak, presented a new combination of alleles: *ctxB*-7/*tcpA* ^CIRS^/*rstR* ^ElTor^. Interestingly, a strain carrying same combination of *ctxB*-7 and *tcpA* ^CIRS^ alleles, as the profile identified in this work, was characterized causing cholera in Cameroon and Haiti in 2010 [17]. Strains from the 2010 Bauchi outbreak in North Central Nigeria and the 2010 Ile-Ife outbreak, both evaluated in this study, were also predominantly of the *ctxB*-7 and *tcpA* ^CIRS^ genotype. Therefore it is likely that these four genotypes were co-circulating in the Chad basin at that time [8].

In the Quilici *et al* study [8], *V. cholerae* strains showed intermediate susceptibility to chloramphenicol, reduced susceptibility to ciprofloxacin and resistance to trimethoprim/sulfamethoxazole, sulfonamides and nalidixic acid, and the *gyrA* ^Ser83Ile^/*parC* ^Ser85Leu^ alleles related to quinolone resistance. This same resistance phenotypic and genotypic profile was found in isolates from all three outbreaks represented in this study (Table 1). Moreover, we found that the 2009/10 O1 Nigeria strains carried *sul2*, *dfrA1* and *floR* genes, conferring resistance to sulfonamides, trimethoprim, and intermediate susceptibility to chloramphenicol, respectively, and *strA/B*, which confers to streptomycin resistance. These genes have been associated with integrative and conjugative elements (ICEs) and the 2009/10 O1 Nigeria strains were positive for the SXT integrase, indicating that these strains harbor an ICE. ICEs have been identified in *V. cholerae* strains from Ghana [24], Kenya [36], Mozambique [37] and Angola [7]. In contrast to the 2009/10 isolates, the Nigeria strains from the beginning of the 7^th^ cholera pandemic had the canonical El Tor allelic profile *ctxB*-3/*tcpA* ^ElTor^/*gyrA* ^Ser83^/*parC* ^Ser85^ and lacked most of the resistance genes.

Interestingly, as reported elsewhere [38], we found that a lineage of *V. cholerae* non-O1/non-O139, lacking the major virulence determinants and the resistance phenotype described above, but harboring a set of virulence associated genes was co-circulating with atypical El Tor strains being recovered from patients with cholera-like disease in Bauchi, Nigeria in 2010. This outbreak followed the Maiduguri outbreak and were accompanied by a slightly improved public engagement response. It is probable that individuals with cholera-like disease are more likely to present to health facilities when they have received information about an on-going cholera outbreak. Our findings suggest that non-O1/non-O139 *V. cholerae* may be important causes of endemic diarrheal disease and outbreaks. Recovery of a similar strain from water tested in Ile-Ife supports this possibility.

Population structure of clinical *V. cholerae* strains can be influenced by epidemiological factors like transmission dynamics, clonal expansion during epidemics, human travel, and selective pressure from antimicrobial drugs. The data in our study showed that recent outbreaks in Nigeria are caused by multidrug resistant atypical El Tor O1 strains, which are reportedly highly virulent [8], [39]–[42], a common worrisome trend in the current cholera outbreaks around the world. The data suggest that guidelines for managing and containing cholera outbreaks in Nigeria (which include, in addition to rehydration, using the antimicrobials trimethoprim, and more recently ciprofloxacin) need to be urgently revised. The data also show that *V. cholerae* non-O1/non-O139 strains were involved in outbreaks in Nigeria suggesting that interventions to control epidemic cholera such as improvements in water supplies and sanitation, as well as vaccination, may result in broad gains.
